# Contradictory individualized self-blaming: a cross-sectional study of associations between expectations to managers, coworkers, one-self and risk factors for musculoskeletal disorders among construction workers

**DOI:** 10.1186/s12891-016-1368-1

**Published:** 2017-01-10

**Authors:** Jeppe Zielinski Nguyen Ajslev, Roger Persson, Lars Louis Andersen

**Affiliations:** 1 National Research Centre for the Working Environment, Lersø Parkalle, 105, 2100 Copenhagen, Denmark; 2 Department of Psychology, Lund University, SE-22100 Lund, Sweden; 3 Division of Occupational and Environmental Medicine, Lund University, SE-22185 Lund, Sweden; 4 Department of Health Science and Technology, Physical Activity and Human Performance group, Aalborg University, Aalborg, Denmark

**Keywords:** Agency, Construction work, Management, MSD (musculoskeletal disorders), Occupational health and safety management (OHS), Pain, Physical exertion, Quantitative method

## Abstract

**Background:**

Within work sociology, several studies have addressed construction workers’ practices of masculinity, class, economy, safety risks and production. However, few studies have investigated room for agency in relation to bodily pain or musculoskeletal disorders and even fewer have made a quantitative approach. Accordingly, by means of a questionnaire, we examined the association between construction workers’ room for agency and physical exertion, bodily and mental fatigue, and lower back pain.

**Methods:**

A total of 481 Danish construction workers who responded to a multifaceted questionnaire were included. Drawing on previous studies and a Foucauldian inspired concept of agency, agency was quantified through specially crafted questions and examined in relation to established measures on physical exertion, physical and mental fatigue and pain in the lower back. Associations were tested using analyses of variance (general linear models) and controlled for age, gender, job group, lifestyle and depression.

**Results:**

When asked about options for agency reducing the burden of work, few workers believed themselves to be prime agents of such practices. When asking about their view on performing alternative agency implying caring for the body, 39–49% expected negative reactions from management, and 20–33% expected negative reactions from colleagues. In contrast, only 13–18% of the participants stated that they would give a negative reception to such alternative practices.

Using the expected reception outcomes (positive, neutral, negative) to alternative practices as predictors, the statistical regression analyses showed that negative expectations to management were associated with higher levels of physical exertion 0.62 (95% CI = 0.14–1.09) (scale 0-11), bodily fatigue 0.63 (95% CI = 0.22–1.04), mental fatigue 0.60 (95% CI = 0.07–1.12), and low back pain 0.79 (95% CI = 0.13–1.46) (scales 0-10).

**Conclusion:**

In our study, construction workers answered questions about work and MSD. The answers indicated a contradiction between perceived responsibility and room for agency.

Based on the study, a number of target areas could fruitfully be addressed in aiming to reduce MSD among construction workers. To change workers’ expectances to the reception of lowering work pace if needed to take care of the body, their expectances to the reception of sickness absence as a result of pain, of discussing physical exertion in work and of demanding appropriate technical assistive devices are such examples. Our results emphasize that management plays an important role in this.

## Background

A number of studies within the field of sociology of work have drawn attention to construction worker’s practices of masculinity, class, economy, safety risks and production. However, few studies have made explicit reflections on these practices in relation to experiences of bodily pain or the high number of workers suffering from musculoskeletal disorders (MSD). Yet MSD is a socio-economic challenge in construction work [1, 2], among blue-collar workers [3] and in contemporary society in general [4, 5]. Knowledge on workers’ practices could contribute with new insights on how to take preventive action.

Based on studies in different Western construction industries, scholars such as Applebaum [6], Gherardi and Nicolini [7], Paap [8], Thiel [9] and Ajslev et al [10] all found that strength, endurance and self-reliance are perceived as positive identity signifiers. Indeed, there is a general agreement that these characteristics are of central importance for maintaining hierarchical positioning in the professional work gang, which is a central unit of organization in most construction work. In addition, strength, self-reliance and endurance are also characteristics valued by employers and local management. In fact, the display of these working-class masculine characteristics is often implicitly valued higher than the obligation to contractual agreements or legislation among workers, site managers and employers. Furthermore, Gherardi and Nicolini [7] and Paap [8] have in their sociological and ethnographic studies identified a tendency among both construction workers and employers to individualize blame for ‘risky behaviour’ rather than emphasizing organizational or collective bases for accidents. This view is confirmed by other studies showing that construction workers often share the common belief that they individually through their work practices are the main agents of exposing themselves to risk factors for developing pain, as they choose to lift heavy loads, work fast and hard and to disregard health recommendations [10].

From a sociological point of view, however, construction workers’ ways of performing work in more or less physically exerting ways and their ways of handling pain, will in some degree be delimited by the socially imbedded expectations on worker conduct and demands for maintaining a positive social identity. According to social theory, the decisions which people make about which actions to take in particular situations is highly, but not exclusively, influenced by their interpretations of what other people will appreciate. This dependency on other people’s presumed reactions is further strengthened if these other people occupy relatively more powerful positions in relation to the person in question (i.e. the agent) [11–14]. In any event, when workers are subjected to the realities of work including time pressure, economic and productive demands in a culture that expects a conformity to masculine working-class characteristics [7, 8, 10], it is appropriate to raise the question of construction workers’ room for *agency*.

This is especially the case in view of the commonness of self-organizing work gangs within the industry as well as a number of studies that all have identified problems with the extant organization of construction work. For example, Baarts described that the projecting and planning of work reminded of a poorly planned ‘trial and error’ approach [15]. In addition, Paap noted that workers felt they were expected to do whatever it takes to complete work as fast as possible without too many complications, thus lowering their demands of technical assistive devices, safety measures or influence on planning [8]. Likewise, Westgaard and Winkel [16] describe a tendency towards the use of technical assistive devices to be applied only if their use increases productivity. As these studies underline, practices in construction seem to urge workers towards performing work and identity in ways that (re)configure and consolidate their identities as strong, enduring, independent, working class men. For this reason, it seems warranted to focus on agency and how the workers’ practices are related to the ways in which they handle physically exerting work tasks and pain.

In contemporary social research, the term agency is widely employed, in more or less explicated forms as the ways in which subjects (people) engage with the world and themselves. Much has been said on agency by many scholars e.g. [17–20]. Acknowledging this, we have for the present purposes adopted a Foucauldian inspired view on agency. This entails understanding agency as the ways in which participants adopt strategies for modifying their degrees of freedom in different situations e.g. situations of school, work, family life, economy, ect. Relations of power do not necessarily pose very narrow limits for socially acceptable conduct, but may take the form of a more direct domination where economic, political or other means block out most alternative forms of practice, while not being fixed indefinitely [13]. Following this, we employed ‘*room for agency’* as an analytical concept and made it part of a research study on Danish construction workers. However, since perspectives on agency and research on construction worker culture generally have been produced through qualitative research, we decided to conceptualize agency into a novel quantitative approach. Accordingly, based on earlier qualitative studies and on our knowledge of construction work, we developed questions that aimed to assess the construction workers’ room for agency in relation to their possibilities for reducing physical exertion, a known risk factor for MSD [21].

In addition, we also created questions that addressed the construction workers’ options for adopting an *alternative agency* and their expectations on how these forms of alternative agencies would be received by colleagues and management. Responses to these questions were subsequently analyzed in relation to known risk factors for developing MSD in the shape of physical exertion, physical and mental fatigue and lower back pain.

## Objectives

The purpose of the present study was thus to 1) examine workers’ room for agency in relation to their possibilities for reducing physical exertion, and 2) to examine the association between construction workers’ expectations to the reception of alternative agencies and physical exertion, fatigue and pain.

## Methods

### Study design

This study is a cross-sectional questionnaire study that was part of a research project that examined working conditions among carpenters, scaffolders, bricklayers, and concrete workers in Denmark. These four groups were selected since they were the primary focus in the broader research programme funded by the Danish Ministry for Research and Innovation and Construction Industry’s Health and Safety Service (CIHSS). The CIHSS is financed by trade unions and employer associations in the Danish construction industry and assists construction companies, safety representatives, and employees in developing and ensuring safe and healthy working conditions.

### Setting

All data was collected between June and December 2013 at worksites, and by means of written questionnaires. Participation was voluntary and participants were eligible for a compensation of maximum EUR 20 (either cash or a gift (i.e. a bottle of wine)).

### Participants

A total of 640 Danish construction workers employed as carpenters, scaffolders, bricklayers, and concrete workers were invited to participate in a questionnaire survey. Of these, 519 (81%) returned the questionnaire. The majority of the present participants have been examined in a study focusing on analyzing the association between wage system and risk factors for musculoskeletal disorders [22].

Participant characteristics can be found in Table 1. The recruitment of respondents was performed in collaboration with the CIHSS. Recruitment for the survey is inspired by Bent Flyvbjerg’s ideas on best case studies [23], as companies and workers in contact with the CIHSS are generally known to be more interested and engaged in health and safety issues than many others in the industry (based on experiences from unions, employers association and CIHSS consultants). Following this line of thought, we can expect the room for prioritizing bodily wellbeing to be better among our respondents than average for the industry.

**Table 1:** Participant characteristics and items 1, 2 and 4

Participant characteristics	Total data				
Number of participants	481				
profession(N)					
Bricklayer’s laborers^β^	23				
Bricklayer	80				
Concrete workers	155				
Scaffolders	63				
Carpenters	160				
Age (Mean years (SD))	39,9 (12.5)				
BMI (Mean kg/m2 (SD))	26.2 (3.6)				
Smokers (%)	177 (38%)				
Depression(%)	31 (6.8%)				
Item 1			Item 2		
*In your opinion, who has the main responsibility for the physical exertion you undertake during work?*				*Do you have the opportunity to reduce the physical strain you experience during work?*	*N* = 472
My-self	312 (65%)			in a very high/high extent	64 (14%)
My colleagues	29 (6%)			to some extent	260 (55%)
My employer	190 (40%)			in a poor/very poor extent	148(31%)
Building material producers/suppliers	55 (11%)				
The construction industry in general	168 (35%)				
Item 4					
1: *How do you think your management will perceive the following?*		positive reception	of no consequence	negative reception	total
		N (%)	N (%)	N (%)	N
that someone lowers work pace to better take care of their body		75 (16%)	151 (31%)	237 (49%)	463
that someone turns in sick because of pains or soreness		81(17%)	194 (40%)	185 (39%)	463
that someone complains about the physical strain in work		88 (18%)	185 (39%)	186 (39%)	459
that someone will only work if the appropriate technical assistive devices are present		94 (20%)	135 (28%)	239 (48%)	459
2: *How do you think your colleagues will perceive the following?*		positive reception	of no consequence	negative reception	
		N (%)	N (%)	N (%)	N
that someone lowers work pace to better take care of their body		116 (24%)	188 (39%)	161 (33%)	465
that someone turns in sick because of pains or soreness		120 (25%)	237 (49%)	104 (22%)	461
that someone complains about the physical strain in work		136 (28%)	229 (48%)	97 (20%)	462
that someone will only work if the appropriate technical assistive devices are present		167 (35%)	171 (36%)	118 (25%)	456
3. *How would you yourself perceive the following?*		positive reception	of no consequence	negative reception	
		N (%)	N (%)	N (%)	N
that someone lowers work pace to better take care of their body		226 (47%)	150 (31%)	85 (18%)	461
that someone turns in sick because of pains or soreness		215 (45%)	186 (39%)	61 (13%)	463
that someone complains about the physical strain in work		245 (51%)	157 (33%)	61 (13%)	463
that someone will only work if the appropriate technical assistive devices are present		262 (55%)	135 (28%)	64 (14%)	461

*SD* Standard deviation

^β^Bricklayer’s laborer’s in Danish construction work are workers who make sure bricklayers have all the materials they need, that the work site is ready to work, clean and tidy as well as plan work ahead. This is a very traditional organization of bricklaying

### Variables

#### Predictors: room for agency

Four items on agency were developed. The items were based on prior qualitative interviews with construction workers and entailed both forced choice and open-ended response alternatives [10].

### Item 1: responsibility for physical strain

The first item read: “*In your opinion, who has the main responsibility for the physical strain you undertake during work?*“, and was responded to on a five-step categorical scale: myself, my colleagues, my employer, building material producers/suppliers, the construction industry in general. Multiple answers were allowed. Results are displayed as frequencies and percentages of total answers in Table 1.

#### Item 2: opportunity to lessen physical strain

The second item read: “*Do you have the opportunity to lessen the physical strain you experience during work?”* and was responded to on a five step scale: 1 = to a very high extent, 2 = to a high extent, 3 = to some extent, 4 = to a small extent, 5 = to a very small extent. For analysis purposes, these items were trichotomized into 1 = in a high/very high extent, 2 = in some extent, 3 = in a poor/very poor extent. Results are displayed in Table 1.

#### Item 3: How to reduce physical strain

The third item entailed an open-ended response and read: “*How can the physical strain, you experience in work, be reduced?* These responses were thematized into categories, not leaving out any answers, but clustering all answers of very similar meaning. Data on this item are presented in Table 2 as frequencies in each category.

**Table 2:** ^a^How to reduce physical exertion in work

How can the physical strain, you experience in work, be reduced?	N
More/better assistive devices	140
Staying at home/no clue/it can’t be done	53
Better planning/projecting/worker influence	33
Lighter, easier handled materials	30
More use or availability of crane	24
Helping each other more	19
Better timeframes/more time/less stress and time pressure	18
Better lifting technique/taking care while lifting	16
Better usage of assistive devices	16
Stricter demands from work authority	7^b^
Forbidding piece rates/better prices so we don’t work so hard	6
Reduced working hours	3
Learning to say no to too hard tasks	3

^a^The following perspectives was left out of the table as they were only mentioned once (which of cause does not make them irrelevant): better descriptions of work, equal distribution of tasks in the gang, companies won’t pay for it, economy, accept from the companies, new thinking

^b^Seven workers propose stricter demands from working authorities as a solution. Whether this would work is a discussion falling outside the scope of this article, however the proposal does not include particular worker agency

#### Item 4: Expected reactions to alternative practice

The fourth item concerned expectations and strived to capture the importance of displaying socially/organizationally acceptable behavior as described in earlier research [7, 8, 10, 15]. The item contained three questions with four statements each, thus generating in total 12 sub-items. The three questions related to what we call ‘*imagined situations*’ and asked about how respondents expected reactions on alternative practices to be. These were alternative practices that presumably could lower physical deterioration, but which we due to our prior knowledge believed to be somewhat controversial and likely to be questioned. The first of these question read “How do you think your management will perceive the following” and pertained to the following four alternative practices “(a) that someone lowers the work pace to take better care of their body; (b) that someone calls in sick because of pain or soreness; (c) that someone complains about the physical strain in work; (d) that someone will only work if the appropriate technical assistive devices are present”. The second and third question repeated this question by exchanging the word “management” with “colleagues” and “you yourself”. All three questions and the four imaginary situations were answered on 5-point scales: 1 = very positive reception, 2 = positive reception, 3 = this will be of no consequence, 4 = negative reception, 5 = very negative reception. Frequencies and percentages on this question are displayed in Table 3.

**Table 3:** Associations between expectations to context and risk factors for MSD

		Physical Exertion (scale 0-11)	Bodily Fatigue (scale 0-10)	Mental Fatigue (scale 0-10)	Low Back Pain (scale 0-10)
Managers	*Neutral (reference)*	*5.97 (5.41–6.53)*	*5.14 (4.65–5.62)*	*3.50 (2.87–4.13)*	*3.68 (2.91–4.45)*
	Negative	**0.62 (0.14–1.09)**	**0.63 (0.22–1.04)**	**0.60 (0.07–1.12)**	**0.79 (0.13–1.46)**
	Positive	**-0.99 (-1.65 - -0.33)**	-0.19 (-0.75–0.38)	-0.18 (-0.90–0.54)	-0.12 (-1.02–0.78)
Work Gang	*Neutral (reference)*	*5.78 (5.22–6.34)*	*5.12 (4.63–5.6)*	*3.84 (3.21–4.46)*	*3.74 (2.96–4.52)*
	Negative	0.12 (-0.43–0.68)	**0.55 (0.07–1.02)**	-0.22 (-0.84–0.39)	0.47 (-0.31–1.25)
	Positive	0.08 (-0.47–0.63)	-0.05 (-0.51–0.42)	-0.37 (-0.97–0.23)	0.01 (-0.74–0.77)
Individual Worker	*Neutral (reference)*	*5.78 (5.23–6.34)*	*5.13 (4.64–5.61)*	*3.51 (2.89–4.13)*	*3.60 (2.82–4.37)*
	Negative	-0.07 (-0.78–0.65)	0.32 (-0.29–0.93)	0.10 (-0.68–0.88)	0.35 (-0.64–1.34)
	Positive	0.25 (-0.23–0.73)	0.16 (-0.25–0.57)	0.29 (-0.25–0.82)	0.58 (-0.11–1.26)

Differences of least square means (95% confidence intervals) for physical exertion, bodily and mental fatigue, and low back pain in relation to negative and positive expectations, respectively, relatively to neutral expectations for managers, gang and individuals workers, respectively. The least square mean value of neutral are provided in *italic* for reference. All outcomes are mutually adjusted for each other in addition to age, job group, lifestyle factors (smoking and BMI), and depression. Significant differences from neutral are marked in bold

For further statistical analysis, the average value was calculated of each of the four questions for 1) management, 2) colleagues, and 3) individual workers, respectively. These were normalized on a scale of 0-100, where 0 is completely negative and 100 is completely positive. Subsequently, we defined 0-40 as negative, >40 to < 60 as neutral, and 60–100 as positive.

### Outcomes: physical exertion, fatigue and pain

#### Physical exertion

The participants’ degree of perceived physical exertion (PE) was measured by the Borg CR10 scale [24, 25]. The question read “In general, how physically exerting do you perceive your current work to be?” The response scale had 16 steps, some of which were verbally anchored: 0 = not at all, 0.3; 0.5 = extremely weak; 1 = very weak, 1.5; 2 = weak, 2.5; 3 = moderate; 4 = somewhat strong; 5 = strong, 6, 7 = very strong; 8, 9, 10 = extremely strong; and 11 = maximal exertion.

#### Fatigue

Fatigue was assessed through the question: “*How fatigued are you after a typical work day?”* This item has previously been used in the validation of self-reported physical exertion [26]. The question was responded in relation to two different bodily locations: (i) in general and (ii) in the head. Both items were responded to on a five-step scale indicating the degree of perceived fatigue: 1 = not fatigued, 2 = a little fatigued, 3 = somewhat fatigued, 4 = fatigued, and 5 = exhausted. These scales were recalculated into a 0-10 scale to improve comparable interpretation with the other scales.

#### Pain

Regional pain intensity in the lower back (LBP) was assessed by asking on an eleven-point scale ranging from ‘0’ (no pain at all) to ‘10’ (worst possible pain). The scale was previously employed by Andersen et al in a study relating pain to long-term sickness absence [3].

### Statistical analysis

Data for the first two tables are presented as unadjusted frequencies and point prevalences.

Table 3 presents results from the statistical analyses of variance using general linear models (Proc GLM, SAS version 9.4). Type 3 sums of squares, F-values (Mean Square of model / Mean Square of error) and corresponding *P*-values were obtained. Physical exertion, bodily fatigue, mental fatigue, and low back pain, respectively, were separate outcomes (dependent variables), i.e. they were not included in the same statistical model. Explanatory (independent) variables for each of the four outcomes were imagined expectations with the variables for managers, colleagues and oneself included in the same model, i.e. they were mutually adjusted. All analyses were further controlled for age, job group, lifestyle factors (smoking and BMI), and depression. These control variables were selected because they potentially could act as confounders in the relation between the explanatory variables and the outcomes. Outcomes are reported as least square means (for neutral expectations) and differences of least square means (for positive and negative expectations) (95% confidence intervals). Cases with missing data were excluded from analysis.

## Results

**Fig. 1:**
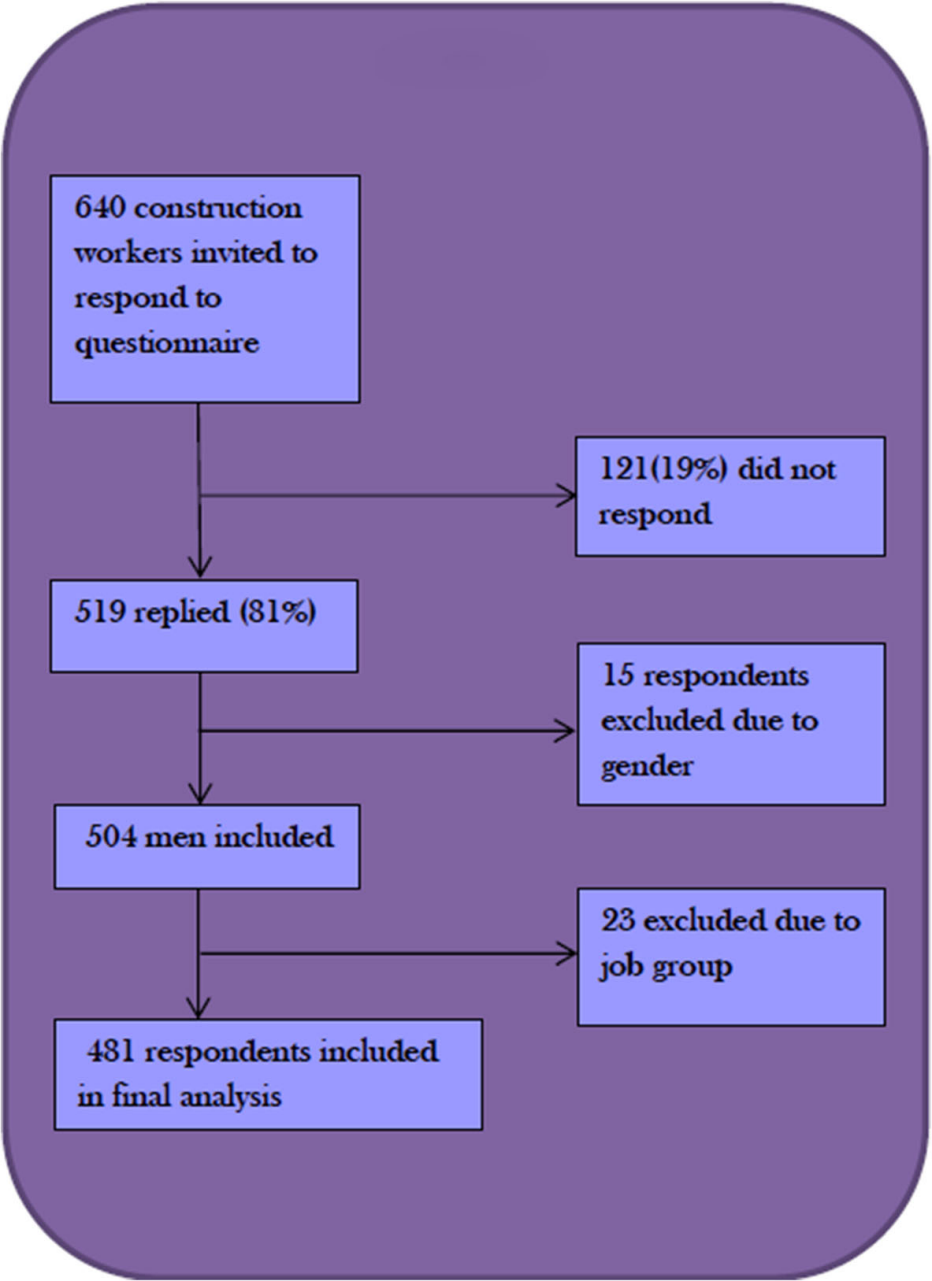
Flowchart

Three women and 12 persons who provided no information on gender were excluded as was participants not working within the four job categories mentioned above. The final study sample thus comprised 481 male construction workers with a mean age of 39.9 years (SD 12.5 years). Figure 1 presents the flowchart of the study, participant characteristics are displayed in Table 1.

### Room for agency: Agency as responsibility for physical exertion

Table 1 shows that some 65% of the respondents perceived themselves as bearing the main responsibility for their own physical exertion. Only 14% of the workers perceived having a high, or very high, opportunity to reduce their physical exertion, while 55% reported to have some extent of opportunity to reduce physical exertion.

### Room for agency: Agency as alternative ideas for reducing strain

In total, 320 of the 481 participants (67%) responded to the open-ended question regarding their own possibilities to reduce strain, generating a total of 374 suggestions. The suggestions were grouped into 13 themes. The three most common themes were “more and better technical assistive devices” (*n* = 140), “staying at home/no clue/it cannot be done” (*n* = 53), and “better planning/projecting/worker influence” (*n* = 33).>

### Room for agency: Alternative agency and outcome expectancy

Table 1 shows that in response to the three questions on outcome expectancy in relation to the “imaginary situations”, we observed that circa 39 - 49% of the participants believed that the managers would display a negative reaction if someone would lower his work pace, turn in sick, complain about physical strain or demand technical assistive devices (Table 1). The corresponding figures for positive expectancies and neutral expectancies were 16–20% and 28–40%, respectively. Furthermore, circa 20–33% of the participants equally displayed negative expectations concerning the colleague’s presumed reception to the similar situations. The corresponding figures for positive expectancies and neutral expectancies were 24–35% and 36–49%, respectively. When turning the question on its head, and asking about their own perception and valuing of other people lowering the work pace, turn in sick, complain about physical strain or demanding technical assistive devices, between 13 and 18% expected to give a negative reception. The corresponding figures for positive expectancies and neutral expectancies were 45–55% and 28–39%, respectively.

### Associations between outcome expectancies to alternative practices and MSD risk factors

The regression analysis between outcome expectations associated with the alternative practices in relation to the “imaginary situations” and risk factors for MSD revealed associations between the workers’ expectations to the management’s reception of alternative practices and reports of physical exertion, bodily and mental fatigue and pain in the lower back (Table 3). Indeed, negative expectations on managers reception of alternative practices were consistently associated with higher levels of physical exertion 0.62 (95% CI = 0.14 - 1.09) (scale 0-11), bodily fatigue 0.63 (95% CI = 0.22 to 1.04), mental fatigue 0.60 (95% CI = 0.07 to 1.12), and low back pain 0.79 (95% CI = 0.13 to 1.46) (scales 0-10). By contrast, positive expectation to managers was associated with lower levels of physical exertion -0.99 (95% CI = -1.65 to -0.33). Turning to the association between the workers’ expectations on their own reaction to colleagues’ practices of alternative agencies, we observed no associations for any of the outcome measures. Likewise, expectations to the reception of alternative practices from colleagues in the gang, does not seem to have particularly strong associations to our investigated risk factors for MSD. Of the four outcomes, only bodily fatigue was worsened by negative expectations with a difference relative to neutral expectations of 0.55 (95% CI = 0.07 - 1.02).

## Discussion

In this article, we conceptualized room for agency in a quantitative manner as a concept allowing for investigation of options for performing alternative practices in relation to physical exertion in work. We investigated this conceptualization in a cross-sectional study among construction workers in Denmark.

### Options for alternative agency among construction workers

Similar to earlier qualitative studies [8, 10], we observed that a large part of workers reported themselves as being mainly responsible for the physical exertion they undertake. However, a minority of the workers report a real opportunity to reduce this exertion (Table 1). Thus, the initial part of our analysis severely questions the notion that workers can be held individually responsible for the development of work-related pains, as actually being accountable would imply opportunity to change the conditions.

In the second part of our analysis that aimed to explore agency in terms of alternative suggestions for practice (item 3, Table 2), we observed that workers came up with relatively few suggestions on how to reduce physical exertion within their work practice. At the same time, the bulk of suggestions reflect a functional agency that is in conformation with common practices and beliefs within construction work. For example, the belief that technical assistive devices alone can reduce physical exertion or that the reduction of physical exertion is impossible. Yet, the uncritical belief that technical assistive devices can solve most problems appears unlikely on the basis of extant knowledge [10, 16].

When asked about the expected reactions to the imagined situations of alternative agency (Table 1), a large proportion of workers reported negative expectations towards managers’ reception of such agencies (39–49%). A somewhat smaller part reported negative expectations towards colleagues’ reception of alternative practices (20–33%). Although we had anticipated negative expectations to the reception of these alternative practices, it is important to observe the workers’ relative weighing of these expectations.

As we analyze the relation between the expectations linked to alternative agencies and physical exertion, physical and mental fatigue and back pain, we find fairly clear patterns. Particularly, negative expectations to managerial acceptance of alternative practices were associated with higher levels (negative) of MSD risk factors. This is in agreement with earlier research pointing to the notion that managerial support is very important to obtain successful occupational health and safety practices in the construction industry [27, 28]. In addition, O’Donnell et al reported similar patterns among workers with managers who showed low support for work-family balance. These workers also reported higher levels of employee-reported pain [29]. Also, Munir et al showed that employers’ support for handling chronic diseases at work is important for appropriate symptom management among workers [30]. As such, other studies have shown the important role of managers in relation to pain and diseases. The particularly interesting part of our finding is the focus on workers’ expectations to managers and their association to risk factors for MSD.

That workers who need most to take care of their bodies are the ones that position themselves as being most limited, poses a significant challenge to prevention of MSD among construction workers. Accordingly, when a worker feels pain, his expectations to managers’ reactions are likely to make him feel and believe that the options to take care of his bodily wellbeing are limited. Thus, managers seem to play a very important role in creating room for agency.

However, the observed lack of room for alterative agencies make sense in a socio-material organizational context where workers are expected to behave like ‘real men’ and because of the time-pressure and fierce levels of competition that both workers and employers experience [7, 8, 10]. What seems to be a matter of choice between taking care of the body during work (being responsible) or not, may be a choice of keeping a job.

It also seems appropriate to compare our results to earlier studies investigating psychosocial factors in the working environment in relation to MSD. In this, the job-demand and -control model (JDC) [31, 32] has been widely accepted and employed, although in various formats. In a review of the association between upper extremity MSD and psychosocial factors at work, Park and Jang concluded that low decision latitude was associated with numbness of the upper extremities; that control over time was associated with neck symptoms and that work demands was associated with neck and shoulder symptoms [33]. Within a similar frame of reference, Eltayeb et al. also identified job demands and hours of working time as predictors for musculoskeletal symptoms in office workers [34]. Also, Smith et al. showed that having a high strain (eg experiencing high job demands and low job control) and passive job (eg low job demands, low job control) increased the odds-ratio for experiencing shoulder symptoms [35]. In the light of these findings, it is no surprise that expectations to managerial reception of alternative practices are associated with risk factors for MSD.

To our surprise, the same pattern is not clear regarding expectations to colleagues’ acceptance of alternative practices. This somewhat contrasts the findings of Ariens et al. who found that low social support from coworkers increased the relative risk of neck pain [36]. This can be interpreted through the notion that power relations can assume the form of a more direct domination, where e.g. political or economic means block out alternative practices [13]. Presumably employers and managers are in a stronger position to affect employment and economy. Anyhow, ruling out collegiate acceptance of alternative practices as an important issue based on this study would not be wise, as negative expectations to colleagues was associated with increased bodily fatigue and also higher (though non-significant) levels of lower back-pain (Table 3).

Our scales are somewhat comparable to the questions proposed to evaluate decision latitude and job demands in the JDC model [31]. The main difference is that our *imagined situations* are more closely contextually developed to target specific characteristics relevant to construction work. This makes it possible to suggest more specific target areas for action as suggested by earlier research [37, 38].

Creating a social and organizational environment in which people have room for taking care of their bodies seems to offer a chance of lowering the levels of pain workers eventually experience. Thus, the optimistic side of this analysis is that workers who expect a positive or neutral managerial reception of the alternative practices actually score lower on exertion, fatigue and pain. Showing and facilitating organizational support of lowering/reducing work pace if needed to take care of the body, a positive view of sickness absence as a result of pain, discussing physical exertion in work and the employment of appropriate technical assistive devices are examples of target areas in developing this environment.

Another form of agency displayed in the survey is the one not taking place, or taking place through workers’ silent acceptance and conformation to the physically straining, painful work. For example, only three respondents proposed that reducing working hours would be a solution to lower strain at work. Lowering the number of working hours could be a solution that would lower the physical exertion. Positive results in this direction was shown by Wergeland et al. in a study where reduction of working hours was correlated to reduced pain as well as exhaustion after work among health care workers [39].

### Methodological considerations

The conflicting, almost paradoxical, character of individualized blaming for physical exertion and pain among construction workers is shown in the quantitative material. This suggest a underutilized potential for exploring social-material phenomena, such as the interactions between room for agency, and reports of physical exertion, fatigue and pain through an informed quantitative approach. Further, the decision to treat the empirical material statistically allowed us to assess and illuminate systematic differences, which would have been hard to argue for through qualitative analyses alone. However, the cross-sectional study design does not allow any causal conclusions to be made.

We will, however, implore that investigation of the ‘room for agency’ in relation to whatever subject in case, must be constructed on the basis of intimate contextual knowledge of practices in the field in question. We would in no way encourage people to employ the same questions for investigating expectations to reception of alternative practices as we do. What we will encourage. however, is to employ the same approach in terms of building on the same method for constructing the questionnaire. The ‘imagined situations’ become a form of denaturalizing quantitative approach. Built on imaginary but not far-fetched examples of practices which might reduce physical strain and pain among construction workers, but earlier described to be contradictory to socially and organizationally appreciated practices [8–10], these questions become ways of questioning the currently naturalized ways of handling physical strain and pain in work.

## Conclusion

In our study, construction workers have answered questions about work and MSD. The answers indicate a contradiction between perceived responsibility and room for agency. In particular, workers expecting that alternative practices will be negatively received by management report increased exertion, pain and fatigue relative to workers expecting a neutral or positive reception. Based on the study, a number of examples of target areas could fruitfully be addressed in aiming to reduce MSD among construction workers. To change workers’ expectances to the reception of lowering work pace if needed to take care of the body, their expectances to the reception of sickness absence as a result of pain, of discussing physical exertion in work and demanding appropriate technical assistive devices are such examples. Our results emphasize that management plays a very significant role in this whereas colleagues seem to play a less important role.

## Abbreviations

CI: Confidence interval
CIHSS: The Construction Industry’s Health and Safety Service
MSD: Musculoskeletal disorders
N: Number
OHS: Occupational health and safety
SD: Standard deviation
